# Functional genomics and expression analysis of the *Corynebacterium glutamicum fpr2-cysIXHDNYZ* gene cluster involved in assimilatory sulphate reduction

**DOI:** 10.1186/1471-2164-6-121

**Published:** 2005-09-13

**Authors:** Christian Rückert, Daniel J Koch, Daniel A Rey, Andreas Albersmeier, Sascha Mormann, Alfred Pühler, Jörn Kalinowski

**Affiliations:** 1 International NRW Graduate School in Bioinformatics & Genome Research, Universität Bielefeld, D-33594 Bielefeld, Germany; 2 lnstitut für Genomforschung, Universität Bielefeld, D-33594 Bielefeld, Germany; 3 Lehrstuhl für Genetik, Universität Bielefeld, D-33594 Bielefeld, Germany

## Abstract

**Background:**

*Corynebacterium glutamicum *is a high-GC Gram-positive soil bacterium of great biotechnological importance for the production of amino acids. To facilitate the rational design of sulphur amino acid-producing strains, the pathway for assimilatory sulphate reduction providing the necessary reduced sulfur moieties has to be known. Although this pathway has been well studied in Gram-negative bacteria like *Escherichia coli *and low-GC Gram-positives like *Bacillus subtilis*, little is known for the *Actinomycetales *and other high-GC Gram-positive bacteria.

**Results:**

The genome sequence of *C. glutamicum *was searched for genes involved in the assimilatory reduction of inorganic sulphur compounds. A cluster of eight candidate genes could be identified by combining sequence similarity searches with a subsequent synteny analysis between *C. glutamicum *and the closely related *C. efficiens*. Using mutational analysis, seven of the eight candidate genes, namely *cysZ, cysY, cysN, cysD, cysH, cysX*, and *cysI*, were demonstrated to be involved in the reduction of inorganic sulphur compounds. For three of the up to now unknown genes possible functions could be proposed: CysZ is likely to be the sulphate permease, while CysX and CysY are possibly involved in electron transfer and cofactor biosynthesis, respectively. Finally, the candidate gene designated *fpr2 *influences sulphur utilisation only weakly and might be involved in electron transport for the reduction of sulphite. Real-time RT-PCR experiments revealed that *cysIXHDNYZ *form an operon and that transcription of the extended cluster *fpr2 cysIXHDNYZ *is strongly influenced by the availability of inorganic sulphur, as well as L-cysteine. Mapping of the *fpr2 *and *cysIXHDNYZ *promoters using RACE-PCR indicated that both promoters overlap with binding-sites of the transcriptional repressor McbR, suggesting an involvement of McbR in the observed regulation. Comparative genomics revealed that large parts of the extended cluster are conserved in 11 of 17 completely sequenced members of the *Actinomycetales*.

**Conclusion:**

The set of *C. glutamicum *genes involved in assimilatory sulphate reduction was identified and four novel genes involved in this pathway were found. The high degree of conservation of this cluster among the *Actinomycetales *supports the hypothesis that a different metabolic pathway for the reduction of inorganic sulphur compounds than that known from the well-studied model organisms *E. coli *and *B. subtilis *is used by members of this order, providing the basis for further biochemical studies.

## Background

In many micro-organisms as well as in higher plants the metabolism of sulphur is based on the uptake and subsequent reduction of oxidised, inorganic sulphur compounds, usually sulphate. While this pathway has been studied in detail in the model organisms *Escherichia coli *for the Gram-negative bacteria [1] and *Bacillus subtilis *for the *Firmicutes *(low-GC Gram-positives), almost no data is available for the high-GC Gram-positives of the order *Actinomycetales *with the exception of the functional analysis of *cysH *from *Mycobacterium tuberculosis *[2].

The pathway for assimilatory sulphate reduction and its genetic basis in *E. coli *and *B. subtilis *can be summarised as follows: After uptake through either an ABC-type transporter, in *E. coli *encoded by *sbp *[EMBL:AAC76899] and *cysUWA *[EMBL:AAC75477, EMBL:AAC75476, EMBL:AAC75475] [1] or, in *B. subtilis*, a permease encoded by *cysP *[EMBL:CAB13432] [3], reduction of inorganic sulphate starts with the activation of sulphate by adenylylation, catalyzed by sulphate adenylyltransferase (EC 2.7.7.4), and yielding adenylylsulphate (APS). In *E. coli *this enzyme is a heteromer encoded by *cysD *and *cysN *[EMBL:AAC75794, EMBL:AAC75793] while *B. subtilis *uses the homomeric protein Sat [EMBL:CAB13437] (encoded by *ylnB *) which is known in other organisms to be involved in dissimilatory sulphate reduction [4]. The release of sulphite from APS can occur either by direct reduction through APS reductase (CysH, EC 1.8.4.10) or by APS kinase (CysC, EC 2.7.1.25) mediated phosphorylation and subsequent reduction of the product, phosphoadenylylsulphate (PAPS), by PAPS reductase (CysH, EC 1.8.4.8). *E. coli *has been demonstrated to use the two-step reaction via CysHC [EMBL:AAC75804, EMBL:AAC75792] while *B. subtilis *CysH [EMBL:CAB13431] can also directly convert APS to sulphite [5]. In either case, sulphite is then reduced to sulphide by sulphite reductase (CysIJ [EMBL:AAC75805, EMBL:AAC75806], EC 1.8.1.2) [6]. In addition to the enzymes directly involved in the catalysis, two additional proteins are thought to be necessary for sulphate reduction in *E. coli: *uroporphyrin-III C-methyltransferase (CysG [EMBL:AAC76393], EC 2.1.1.107) and PAPS phosphatase (CysQ [EMBL:AAC77171]). The former is needed for the biosynthesis of the sulphite reductase cofactor siroheme, catalyzed by YlnD [EMBL:CAB13435] and YlnF [EMBL:CAB13437] in *B. subtilis *[7], while the latter is believed to eliminate excess amounts of PAPS which are highly toxic for the cell [1].

In a continuation of our work to elucidate the biosynthesis of L-methionine in the Gram-positive non-sporulating actinobacterium *Corynebacterium glutamicum *[8], the reduction of inorganic sulphur compounds was investigated in order to learn how the reduced sulphur needed for incorporation into L-methionine is formed. Based on the knowledge of assimilatory sulphur reduction and the genes described for the two model organisms *E. coli *and *B. subtilis*, we tried to identify the set of genes involved in the assimilatory reduction of inorganic, oxidised sulphur compounds in *C. glutamicum*. Real-time RT-PCR was then applied to analyse the operon structure of the genes found and to elucidate their regulation in cells incubated with different sulphur sources. Subsequently, RACE-PCR was used to map the transcription start points of the identified transcription units to identify the potential promoters. Finally, comparison with other completely sequenced members of the *Actinomycetales *was carried out in order to learn about the phylogenetic distribution of the assimilatory sulphur reduction genes.

## Results

### Identification of a *Corynebacterium glutamicum* gene cluster possibly involved in assimilatory sulphate reduction by sequence similarity and comparative genomics

In a previous investigation, the whole genome sequence of *C. glutamicum *[9,10] was used to elucidate the pathway for L-methionine biosynthesis [8]. In this study, we extended this work and analysed the genetic basis for the reduction of inorganic sulphate. Using genes known to be involved in the assimilatory reduction of sulphate from *Escherichia coli *[1], *Mycobacterium tuberculosis *[2], and *Bacillus subtilis *[3,7,11] similar coding sequences (CDS) were searched for in the *C. glutamicum *genome sequence.

**Figure 1:**
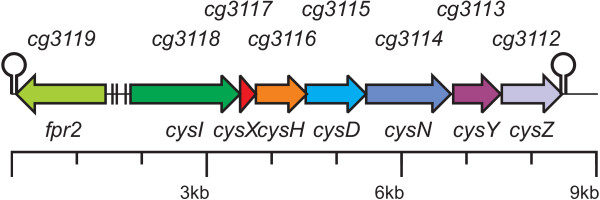
**The *fpr2cysIXHDNYZ* gene cluster in the *C. glutamicum* genome**. Coloured arrows indicate genes that are part of the cluster most probably involved in assimilatory sulphate reduction. Hairpins mark potential rho-independent transcription termination signals predicted by the TransTerm software. Black bars denote binding sites for the transcriptional repressor McbR [15].

This approach led to the identification of a cluster of four CDS (*cg3118, cg3116, cg3115*, and *cg3114 *) in *C. glutamicum *which might be the orthologues of the *E. coli *genes *cysI, cysH, cysD*, and *cysN *(Table 1 and Figure 1), while no genes with high similarity to the *E. coli *genes *cysC, cysJ, sbp *or the *cysPUWA *cluster could be found (data not shown). Likewise, no clear homologues of the *B. subtilis *genes *cysP *or *ylnB *were detected. Closer examination of the genomic neighbourhood of the four candidate genes revealed another three CDS which are located within (*cg3117 *) or directly adjacent (*cg3112, cg3113 *) to the identified cluster of *cys *genes. To determine the boundaries of this cluster, all genes in this genomic region were compared with the genome of the closely related species *Corynebacterium efficiens *[12], using a bidirectional best BLAST hit method [13].

**Table 1:** Properties and sequence similarities of the *C. glutamicum* proteins possibly involved in assimilatory reduction of sulphate

*C. glutamicum*	protein properties					BLAST results^*b *^		
CDS (protein ^*a *^)	length (aa)	pI	mw [kDa]	hit against	in organism^*c *^	description	E-value	identical/positive aa
Cg3112 (CysZ)	309	9.98	32.0	Q8FM69	*C. efficiens*	Conserved hypothetical protein	2e-134	77/88%
				Q81NU8	*B. anthracis*	Putative membrane protein	8e-58	46/64%
				Q813F4	*B. cereus*	Hypothetical membrane spanning protein	4e-57	46/64%
Cg3113 (CysY)	241	5.44	24.7	Q8FM68	*C. efficiens*	Hypothetical protein	1e-80	67/77%
				P61817	*B. megaterium*	Sirohydrochlorin ferrochelatase SirB	5e-16	28/45%
				Q93RW8	*S. coelicolor*	Hypothetical protein SC01858	2e-13	31/43%
Cg3114 (CysN)	433	5.08	46.9	Q8FM67	*C. efficiens*	Putative sulphate adenylyltransferase SU 1	0.0	84/90%
				Q9ADG6	*S. coelicolor*	Sulphate adenylyltransferase SU 1	3e-lll	55/67%
				Q10600	*M. tuberculosis*	Sulphate adenylyltransferase SU 1	1e-104	50/69%
Cg3115 (CysD)	304	5.09	34.3	Q8FM66	*C. efficiens*	Putative sulphate adenylyltransferase	7e-168	95/96%
				Q9ADG5	*S. coelicolor*	Sulphate adenylyltransferase SU 2	2e-123	71/83%
				Q9X5UO	*S. lavendulae*	Sulphate adenylyltransferase SU 2	6e-123	70/82%
Cg3116 (CysH)	261	5.30	28.5	Q8FM65	*C. efficiens*	Putative PAPS reductase	3e-129	86/89%
				Q82L82	*S. avermitilis*	Putative PAPS reductase	7e-52	56/67%
				P71752	*M. tuberculosis*	APS reductase	5e-52	54/69%
Cg3117 (CysX)	82	7.86	9.5	Q8FM64	*C. efficiens*	Hypothetical protein	5e-36	81/89%
				Q82L83	*S. avermitilis*	Hypothetical protein	4e-05	50/57%
				Q9ADG2	*S. coelicolor*	Hypothetical protein SC06101	5e-05	50/57%
Cg3118 (CysI)	561	5.53	63.0	Q8FM63	*C. efficiens*	Putative ferredoxin-nitrite reductase	0.0	91/95%
				Q7WT38	*Streptomyces *sp.	Nitrile/sulphite reductase	0.0	56/70%
				Q82L84	*S. avermitilis*	Putative nitrile/sulphite reductase	0.0	55/71%
Cg3119 (Fpr2)	457	4.88	50.1	Q8FM62	*C. efficiens*	Putative ferredoxin-NADP reductase	0.0	87/93%
				Q8NM28	*C. glutamicum*	NADPH-glutamate synthase beta chain	0.0	74/86%
				Q8FMB5	*C. efficiens*	Putative ferredoxin/adrenodoxin reductase	0.0	75/85%
				FPRA_MYCTU	*M. tuberculosis*	Ferredoxin-NADP reductase FprA	6e-83	39%
				FPRB_MYCTU	*M. tuberculosis*	Ferredoxin-NADP reductase FprB	63e-66	36%

^*a *^by sequence similarity

^*b *^identified by BLASTP similarity search. The protein sequences derived from the *C. glutamicum *candidate CDS were used as queries against the UniProt database

^*c *^*B. = Bacillus, C. = Corynebacterium, M. = Mycobacterium, S. = Streptomyces*

Thereby it could be shown that besides the identified *cys *orthologues, the localisation of *cg3112, cg3113*, and *cg3117 *is also well conserved between *C. glutamicum *and *C. efficiens*, as is that of *cg3119 *while directly up- and downstream of the cluster the syntenous order of genes is interrupted by insertions in *C. glutamicum *(data not shown). The notion that the *cys *gene-cluster extends from *cg3112 *to *cg3119 *is corroborated by the finding that transcriptional termination signals were predicted by the TransTerm software [14] directly behind *cg3112 *and *cg3119 *(Fig. 1). This furthermore suggests that *cg3118 *to *cg3112 *form an operon, providing another hint that also *cg3112, cg3113*, and *cg3117 *are involved in the reduction of sulphate. Additional evidence that the whole cluster is involved in sulphur metabolism was the identification of four binding sites of the transcriptional repressor McbR which controls almost all genes involved sulphur metabolism in *C. glutamicum *[15] in the intergenic region shared by *cg3118 *and *cg3119 *(Fig. 1).

### Bioinformatic analysis of the *Corynebacterium glutamicum* candidate genes possibly involved in assimilatory sulphate reduction

As a first step in the analysis of the eight candidate genes similarity searches against the UniProt and PFAM databases were done. These searches led to a functional assignment for *cg3114 *and *cg3115 *as *cysD *and *cysN *respectively, most likely encoding the two subunits for sulphate adenylyltransferase known from, e.g., *E. coli *(Table 1).

For Cg3118 and Cg3116, the similarity searches indicated possible deviations from the enzymatic functions described for *E. coli *(Table 1): Cg3118 (CysI) from *C. glutamicum*, while moderately similar to CysI from *E. coli*, displays a higher degree of similarity to ferredoxin-dependent sulphite and nitrite reductases (NirA) known from other *Actinomycetales *and plants. In turn, based on a multiple alignment, these sequences cluster much more closely with the ferredoxin-dependent nitrite reductases from plants and cyanobacteria (e.g. Nir from *Synechococcus *sp.) than with CysI homologues of the *a- *and γ-proteobacteria (data not shown). Likewise, Cg3116 (CysH) was found to be much more similar to CysH from *M. tuberculosis *which has been proven to act as APS reductase (EC 1.8.4.10) [2]. Furthermore, all the sequence motifs demonstrated to be necessary for the reduction of APS [16] are present in CysH from *C. glutamicum *(data not shown).

For the proteins encoded by the four novel CDS *cg3112, cg3113, cg3117*, and *cg3119*, no significant similarities to proteins known to be involved in sulphate reduction were detected, and only Cg3119 shows similarities to well characterised proteins (Table 1). The gene *cg3112 *encodes a protein similar to putative membrane proteins of the domain of unknown function family 81 (DUF81), which consists of integral membrane proteins. The notion that Cg3112 is located in the membrane is further corroborated by the TMHMM software which predicts the presence of six transmembrane helices. The possible presence of a signal peptide, detected by SignalP, indicates a Sec-dependent insertion into the membrane (data not shown). Cg3113 is weakly similar to sirohydrochlorin ferrochelatase from *B. megaterium*, indicating a possible involvement in the biosynthesis of the siroheme cofactor of sulphite reductase. The small CDS encoding Cg3117 shows similarities to small hypothetical proteins in several *Actinomycetales*. A multiple alignment of the significant hits revealed the presence of a conserved sequence motif (CPYCx(14,19)WxCxxCxRxF) containing four conserved cysteine residues. They are reminiscent of those found in Fe-S cluster coordination sites. Finally, Cg3119 is highly similar to FprA and FprB, known to act as ferredoxin-NADP reductases in mycobacteria. FprA from *M. tuberculosis *has been studied on the molecular level and is now classified as NADPH-ferredoxin reductase (EC 1.18.1.2), but it is unknown in which biological processes FprA and FprB are involved in *M. tuberculosis *[17,18]. Interestingly, a highly similar paralogue (Cg3049) is present in the *C. glutamicum *genome itself. Based on the well characterised FprA protein of *M. tuberculosis, cg3049 *and *cg3119 *were therefore named *fprl *and *fpr2*, respectively.

### Comparison of the putative sulphate assimilation gene cluster of *Corynebacterium glutamicum* with gene clusters of other members of the actinomycetes

As the cluster of genes possibly involved in sulphate assimilation had been found to be conserved between *C. glutamicum *and *C. efficiens*, the question arose whether the findings for sulphate assimilation in *C. glutamicum *are specific for these two organism or if the cluster and the pathway is conserved among the order of the *Actinomycetales*. Therefore, a search for conserved parts of the cluster in all completely sequenced genomes of the bacterial order as well as in the only partially completed genomes of *Brevibacterium linens *and *Thermobifida fusca *was conducted, using the programs GECKO [19] and TheSEED [20].

This approach revealed that most of the genes found to be involved in sulphur metabolism in *C. glutamicum *are also found in other members of the *Actinomycetales*. Notable exceptions are *C. diphtheriae, Bifidobacterium longum, Leifsonia xyli, M. leprae, Propionibacterium acnes*, and *Tropheryma whipplei*, as in all these organisms the genes needed for assimilatory sulphate reduction are completely absent. All these organisms are known pathogens or commensals of humans, animals, or plants, the most likely explanation is that these bacteria obtain the required sulphur-containing organic compounds directly from their hosts.

**Figure 2:**
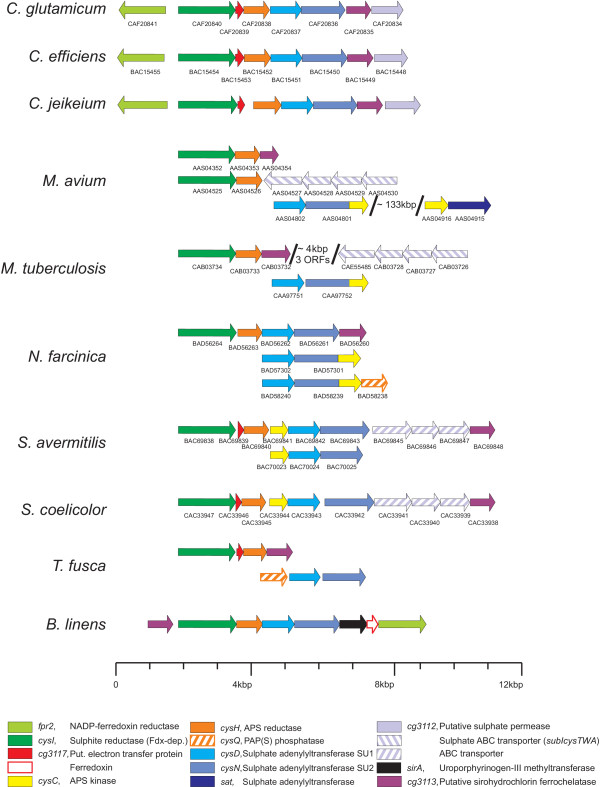
**Conservation of the *C. glutamicum* gene cluster possibly involved in assimilatory sulphate reduction in the Actinomycetales**. Functions were inferred based on sequence similarity from BLAST searches against the UniProt database. Only those genes are displayed that were found to be clustered (at least two adjacent genes possibly involved in assimilatory sulphate reduction).

Concerning the conservation of the cluster, it was found to be almost perfectly conserved in *C. glutamicum *and *C. efficiens *(Fig. 2), as well as in the recently sequenced *C. jeikeium *[21]. In the other members of the *Actinomycetales *that contain the genes needed for sulphate assimilation, these genes are also clustered, but in many cases the cluster is split in two or three parts. In addition, duplications seem to have occurred, e.g., in *M. avium, N. farcinica*, and S. *avermitilis*.

Of the four novel genes *cg3112, cg3113, cg3117 *and *cg3119 *(*fpr2 *), only a *cg3113*-like gene was found in all *Actinomycetales *containing parts of the cluster (Fig. 2), adding additional evidence that it is involved in sulphate assimilation. On the other hand, it proved to be the least conserved on the amino acid level (data not shown).

*cg3112 *seems to be present only in the *Corynebacteriaceae*. This might be due to the fact that in the *Mycobacteriaceae, Streptomycetaceae*, and *N. farcinica *an ABC-type transporter replaces Cg3112.

Fpr homologues are only found to be clustered with other *cys *genes in *Corynebacteriaceae*, but at least one possible homologue is present in all *Actinomycetales *that contain the cluster. Another hint for the involvement in the reduction of sulphate is the co-occurrence of Cg3117 and Fpr homologues lacking a ferredoxin-domain: *M. tuberculosis, M. avium*, and *N. farcinica *all contain a two-domain protein consisting of a ferredoxin-like and a Fpr-like domain. The *Corynebacteriaceae, Streptomycetaceae*, and *T. fusca *all lack such a protein but possess a Cg3117-homologue in the cluster. As the flavoprotein subunit of sulphite reductase CysJ is absent in all *Actinomycetales*, the data from the comparative genomics approach provides circumstantial evidence that the enzymatic equipment for sulphite reduction in the *Actinomycetales *is similar to that found in plants: In these eukaryotes electrons are transferred directly from photosystem I via ferredoxin-NADP reductase and ferredoxin to the ferredoxin-dependent sulphite reductase [22]. For *C. glutamicum*, this leads to the hypothesis that electrons might be transferred from NADPH+H^+ ^by Fpr2 to Cg3117 which in turn delivers them to the ferredoxin-dependent sulphite reductase.

### Characterisation of the *Corynebacterium glutamicum* gene cluster possibly involved in assimilatory sulphate reduction by targeted gene deletion, mutational analysis, and complementation

To determine if the CDS of the identified gene cluster *cg3119-3112 *are involved in the assimilatory reduction of sulphate, deletion mutants of all genes were constructed. Gene-SOEing was used to create fusion products of chromosomal DNA sections of approximately 500 bp length directly up- and downstream of the target gene. The resulting fusion products were cloned into the vector pK18*mobsacB* and the obtained plasmids were used for targeted gene deletion.

The resulting mutants CR018 to CR025 (Table 4) were tested for their ability to grow on a solid minimal medium containing differing inorganic sulphur compounds at 2 mM concentration as sole source of sulphur. Seven of the eight mutant strains CR018 to CR024 were affected in their ability to utilise inorganic sulphur sources. The most common observed phenotype was a complete inability to grow, like strain CR024 (Δ*cysI *) on medium containing sulphate or sulphite, but in some cases, growth was only severely reduced, like that of strain CR023 (Δ*cg3117 *) when utilising oxidised inorganic sulphur compounds (Table 2).

**Table 2:** Growth of *C. glutamicum* mutant strains on different inorganic sulphur sources on solid minimal medium

	deleted	growth^*a *^after 48 h on MMS with addition of 2 mM of				
strain	CDS (gene)	sulphate	sulphite	thiosulphate	sulphide	L-cysteine
WT	/	+	+	+	+	+
CR018	*cg3112 *(*cysZ *)	-	-	+	+	+
CR019	*cg3113 *(*cysY *)	-	-	-	°	+
CR020	*cg3114 *(*cysN *)	-	-	°	+	+
CR021	*cg3115 *(*cysD *)	-	-	°	+	+
CR022	*cg3116 *(*cysH *)	-	-	°	+	+
CR023	*cg3117 *(*cysX *)	°	°	°	+	+
CR024	*cg3118 *(*cysI *)	-	-	-	°	+
CR025	*cg3119 *(*fpr2 *)	+	+	+	+	+

^*a *^compared to the wild-type grown on MMS containing sulphate:

+ denotes wild-type like growth

° indicates a severely reduced growth rate

- represents a complete lack of growth

All four mutants in the potential *cys *gene homologues (*cg3114, cg3115, cg3116*, and *cg3118*) were unable to utilise sulphate as sole source of sulphur while they could still grow on sulphide, thereby confirming that the encoded proteins are involved in the reduction of inorganic sulphur compounds. Surprisingly, the mutants lacking *cysD, cysH*, or *cysN *were also unable to grow on sulphite and displayed a reduced growth rate on thiosulphate. This observation stands in contrast to the findings made, e.g., for *E. coli*, where a loss of these genes does not influence the ability to utilise sulphite or thiosulphate [1].

Of the four novel genes, the mutational analysis provided strong evidence that the three previously uncharacterised CDS *cg3117, cg3113*, and *cg3112 *are involved in assimilatory sulphate reduction (Table 2). The corresponding mutant strains could either no longer grow on MMS containing only sulphate or sulphite (Δ*cg3113 *and Δ*cg3112*) or displayed a severely reduced growth rate (Δ*cg3117*). Therefore, they were named *cysY, cysZ*, and *cysX*, respectively. On the other hand, a loss of *fpr2 *did not influence growth under the tested condition, indicating that the encoded protein is either not involved in sulphate reduction or its function can be replaced, most likely by Fpr1, the product of *cg3049*. Unfortunately, all attempts to verify this hypothesis failed, because deletion or disruption of *fpr1 *was not possible. This indicates that Fpr1 might have another, essential function in *C. glutamicum*.

As the genes *cysI *to *cysZ *might constitute an operon, homologous complementation of the mutant strains CR018 to CR024 (Table 4) was used to verify that the observed phenotypes were due only to the loss of the deleted gene and not caused by polar effects on genes downstream of the deletion. This was done by PCR amplification of the gene in question and subsequent cloning of the product in a suitable expression vector. The resulting plasmids pCR018e to pCR024e (Table 4) were transferred into the corresponding mutant strains and the ability of the plasmid carrying strains to utilize inorganic sulphur sources was tested as described above. In all cases, growth of the mutant strain expressing the missing gene *in trans *was indistinguishable from that of the wild-type, thus ruling out polar effects.

The plasmids pCR020e, pCR022e and pCR024e (carrying *cysDN, cysH*, and *cysI*, respectively) were also used to try heterologous complementation of corresponding *E. coli *mutant strains to get additional evidence for the assumed gene functions. Surprisingly, the complementation of *E. coli *JM221 (*cysD*^-^) and JM246 (*cysI*^-^) with pCR20e, respectively, failed. Only AB462 (*cysC*^-^) and JM226 (*cysH*^-^) carrying pCR022e were able to grow with sulphate as sole source of sulphur, and even these grew only slowly. These results add evidence to the hypothesis that *C. glutamicum *uses either a pathway differing from that in *E. coli *or that the enzymes can only function as a complex in *C. glutamicum*.

### Analysis of the *Corynebacterium glutamicum* gene cluster *fpr2cysIXHDNYZ* by growth tests in liquid culture

Based on the data obtained from the *in silico *and *in vivo *analysis, a hypothesis of the possible functional role of the four novel CDS was formulated, leading to a preliminary model for the pathway for assimilatory sulphate reduction in *C. glutamicum: *The presence of transmembrane domains and the inability of the mutant strain to grow on sulphate and sulphite as sole sources of sulphur indicated that CysZ might be involved in the uptake of these compounds. For CysY, a function in the biosynthesis of the siroheme cofactor of sulphite reductase could be supposed as the phenotype of the CR019 (Δ*cg3113*) mutant strain was indistinguishable from that of CR024 (Δ*cysI*). The small protein CysX might be involved in electron transfer, with the four conserved cysteine residues possibly being part of a Fe-S cluster like those found in ferredoxins. For Fpr2, a function in the reduction of ferredoxins could be assumed, possibly acting on CysX.

In order to substantiate these hypotheses, growth of the mutant strains CR018, CR019, CR023, and CR025 (Δ*cysZ*, Δ*cysY*, Δ*cysX*, and Δ*fpr2*, respectively) was measured via real-time nephelometry in liquid minimal medium on different inorganic sulphur sources at a concentration of 2 mM. From the obtained growth curves, the generation times and the duration of the lag phases were calculated and compared to those determined for the wild-type strain (Table 3).

**Table 3:** Growth characteristics of *C. glutamicum* mutant strains on different inorganic sulphur sources at 2 mM concentration in liquid medium

	generation time^*a *^[h]					lag time^*a *^[h]				
sulphur source	WT	CR018 (Δ*cysZ*)	CR019 (Δ*cysY*)	CR023 (Δ*cysX*)	CR025 (Δ*fpr2*)	WT	CR018 (Δ*cysZ*)	CR019 (Δ*cysY*)	CR023 (Δ*cysX*)	CR025 (Δ*fpr2*)
sulphate	2.1	**nd**^*b,c*^	**nd**	**6.2**	2.2	8.0	**nd**	**nd**	**23.5**	**11.0**
sulphite	2.2	**nd**	**nd**	**4.2**	2.1	8.0	**nd**	**nd**	**16.0**	**11.0**
thiosulphate	3.2	3.2	**nd**	**9.5**	2.9	9.5	**14.0**	**nd**	**37.5**	10.5
sulphide	3.8	3.8	**17.6**	**7.4**	**3.2**	15.0	13.5	16.5	**23.5**	15.5

^*a *^averages, calculated from 18 measurements (3 independent cultivations, 6 replicates per cultivation)

^*b *^significant changes are given in **bold **script

^*c *^nd = not determined due to lack of growth

**Table 4:** Bacterial strains and plasmids

Name	Relevant genotype/information ^*a *^	Source/reference
*E. coli*		
DH5αMCR	F^- ^*endA1 supE44 mcrA thi-1 hsdR17 *λ^- ^*recA1 relA1 Δ *(*lacZYA*-*argF *)*U169 *(Φ 80d*lacZΔM15 *) *gyrA96 deoR *Δ (*mrr-hsdRMS-mcrBC *)	[40]
AB462, CGSC 462	F^- ^*DE *(*gpt-proA *)*64 lacYl galK2 *(Oc)*hisG4 *(Oc) λ^- ^*cysC9 xylA5 mtl-1 thi-1*	CGSC^*b *^
JM226, CGSC 5468	F^- ^*pro-0 lac *^-^*tsx *^-^? *galT47 trp-74 his *^-^*cysH57 xyl argA9999 rpsL *^-^(str^R^) *mal *^-^*ilvA *^-^*mtl *^-^	CGSC, [47]
JM221, CGSC 5745	F^- ^*pro-50 lac- tsx *^-^*galT47 trp-74 his-97 cysD91 argA*^-^*rpsL*^-^-(str^R^) *mat xyl*^-^*ilvA*^-^*mtl*^-^	CGSC, [47]
JM246, CGSC 5747	F^- ^λ^- ^*cysI53*(Am)*IN*(*rrnD-rrnE*)*1 rph-1*	CGSC, [47]
		
*C. glutamicum*		
ATCC 13032	Wild type, Nx^r^	ATCC^*c*^
CR018	Δ*cg3112 *(*cysZ*)	this study
CR019	Δ*cg3113 *(*cysY*)	this study
CR020	Δ*cg3114 *(*cysN*)	this study
CR021	Δ*cg3115 *(*cysD*)	this study
CR022	Δ*cg3116 *(*cysH*)	this study
CR023	Δ*cg3117 *(*cysX*)	this study
CR024	Δ*cg3118 *(*cysI*)	this study
CR025	Δ*cg3119 *(*fpr2*)	this study
CR026	*cg3118*::IS*6100*, pAT6100	this study
		
Plasmids		
pK18*mobsacB*	*sacB, lacZa*, Km^r^, mcs mobilizable vector, allows for selection of double-crossover in *C. glutamicum*	[35]
	mobilizable vector, allows for selection of double-crossover in *C. glutamicum*	
pEC-XK99E	P*trc, lacI*^q^, Km^r ^inducible *E. coli *– *C. glutamicum *– shuttle expression vector	[48]
pAT6100	IS*61 00*, Km^r^	[36]
pCR018d	pK18*mobsacB *carrying *cg3112 del*^*d*^	this study
pCR018e	pEC-XK99E carrying *cysZ *ev^*e*^	this study
pCR019d	pK18*mobsacB *carrying *cg3113 *del	this study
pCR019e	pEC-XK99E carrying *cysY *ev	this study
pCR020d	pK18*mobsacB *carrying *cg3114 *del	this study
pCR020e	pEC-XK99E carrying *cysDN *ev	this study
pCR021d	pK18*mobsacB *carrying *cg3115 *del	this study
pCR022d	pK18*mobsacB *carrying *cg3116 *del	this study
pCR022e	pEC-XK99E carrying *cysH *ev	this study
pCR023d	pK18*mobsacB *carrying *cg3117 *del	this study
pCR023e	pEC-XK99E carrying *cysX *ev	this study
pCR024d	pK18*mobsacB *carrying *cg3118 *del	this study
pCR024e	pEC-XK99E carrying *cysI ev*	this study
pCR025d	pK18*mobsacB *carrying *cg3119 *del	this study

^*a *^r superscript indicates resistance. nx, Nalidixic acid; Km, Kanamycin

^*b *^CGSC; *E. coli *Genetic Stock Center, Yale University, New Haven, CT

^*c *^ATCC; American Type Culture Collection, Rockville, MD

^*d *^the postfix *del *indicates inserts used for targeted gene deletion

^*e *^the postfix *ev *indicates genes preceded by an artificial RBS

This approach confirmed the observations made during growth on solid minimal medium. In the case of CysX, it provided further evidence for the hypothesis that CysX might act like a ferredoxin: The generation time of the mutant strain CR023 (Δ*cysX*) is doubled to tripled (Table 3), indicating that the function of CysX can be partially replaced by another protein, most likely one of the ferredoxins found in *C. glutamicum*. Interestingly, under these conditions strain CR025 (Δ*fpr2*) also delivered a phenotype, namely an increase in the lag phase during growth with sulphate or sulphite of approximately three hours. This provides circumstantial evidence that Fpr2 is indeed involved in sulphur reduction, although its loss can be compensated for.

**Figure 3:**
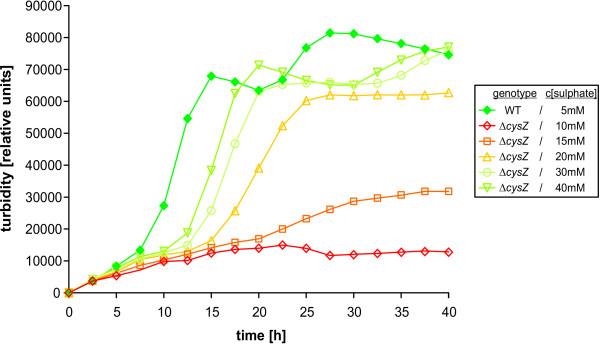
**Growth of the *C. glutamicum* wild-type and the *ΔcysZ* mutant strain in medium with different sulphate concentrations**. Wild-type and mutant strain were grown in liquid minimal medium, cell growth was determined by real-time nephelometry. For each time point the mean of 18 measurements is displayed (3 independent cultivations, 6 parallels per cultivation). The wild-type strain is denoted with filled diamonds, open symbols are used to indicate the mutant strain.

As prior growth tests on solid minimal medium containing high concentrations of sulphate had shown that strain CR018 (Δ*cysZ*) could grow under these conditions (data not shown), additional tests with this strain were performed in liquid culture using varying concentrations of sulphate. These tests confirmed that the mutant strain is unable to grow with sulphate concentrations of less than 5 mM. With increasing sulphate concentration, growth is gradually restored until, at above 30 mM, it reaches almost the level of the wild-type strain (Fig. 3). This concentration-dependent restoration of growth provides strong evidence that CysZ acts as the high-affinity sulphate transporter in *C. glutamicum*. Furthermore it indicates that at least one other transporter for sulphate must exist which is able to function effectively only at sulphate concentrations above 5 mM.

### Transcriptional analysis of the *Corynebacterium glutamicum cys* gene cluster

**Figure 4:**
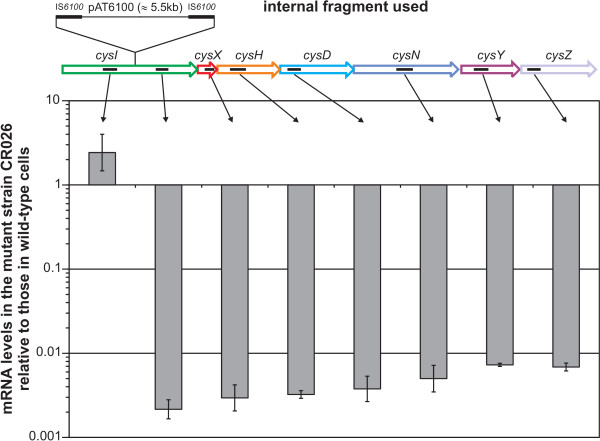
**Comparison of the mRNA levels of the *cys* genes of the *C. glutamicum* wild-type and a *cysI* transposon mutant**. Total RNA was isolated from cells grown in MMS with 1 mM L-cysteine as sulphur source and the relative transcription levels were determined using real-time RT-PCR to quantify the mRNAs of the displayed genes. Small black bars inside the arrows representing the genes indicate the position of the internal fragments amplified in the real-time RT-PCR.

As gene order and the presence of possible transcription termination sites indicated that the genes *cysI *to *cysZ *might form an operon (Fig. 1), real-time RT-PCR was used to verify this hypothesis. To disrupt the hypothetical operon, a transposon mutant in *cysI *(CR026, insertion 997 bp downstream of the *cysI *gene start) was used, as it could be assumed that the insertion of the transposon and the vector with a size of ≈ 5.5 kb would lead to a premature termination of transcription. Using real-time RT-PCR, the mRNA levels of the seven genes in the wild-type and the mutant strain both grown with L-cysteine as sulphur source were compared. The measurements revealed an equally strong reduction of the relative mRNA levels of all seven genes in the mutant downstream of the transposon insertion site (Fig. 4). This observation and the finding that the mRNA level upstream of the insertion is only slightly increased provides strong evidence that *cysIXHDNYZ *indeed form an operon and that no additional promoters downstream of *cysI *exist.

**Figure 5:**
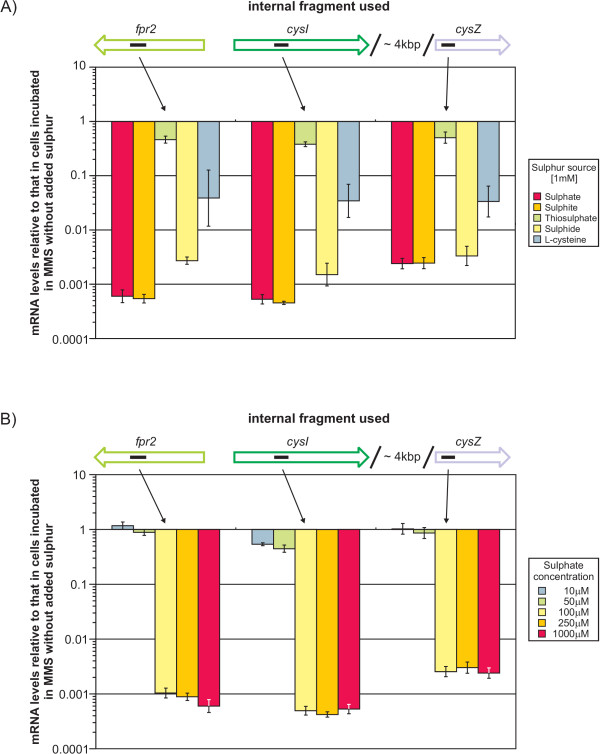
**Change of the *fpr2cysIXHDNYZ* mRNA levels in the presence of different sulphur sources and varying amounts of sulphate**. The relative mRNA levels of the *fpr2*, *cysI*, and *cysZ *genes in cells incubated in MMS with either (A) different sulphur sources at 1 mM concentration or (B) sulphate at varying concentrations were compared to that in cells incubated in MMS without additional S-sources using real-time RT-PCR. Small black bars inside the arrows representing the genes indicate the position of the internal fragments used in real-time RT-PCR.

Real-time RT-PCR was also applied to analyse whether the clustered genes are subject to regulation by either one or more of the inorganic substrates or by the products of assimilatory sulphur reduction (namely sulphide and L-cysteine). Therefore, the mRNA levels of *fpr2, cysI*, and *cysZ *in cells incubated in MMS with different sulphur sources at 1 mM concentration was compared with those in cells incubated in MMS without added sulphur. In all cases with the exception of thiosulphate, a strong simultaneous reduction of mRNA abundance of all three genes in cells incubated with a sulphur source was observed (Fig. 5A). Transcription levels of the genes of the cluster were found to be strongly reduced in the presence of most inorganic sulphur sources while L-cysteine had a much weaker, albeit still significant effect. Surprisingly, the relative transcription levels decreased only marginally in presence of thiosulphate, even at higher concentrations of up to 5 mM (data not shown).

This data clearly proves that the assimilation of sulphur in *C. glutamicum *is regulated by either the substrates or the products of the assimilatory reduction of sulphate. Furthermore, the data indicates that this regulation is different from that found in *E. coli*, as thiosulphate has been shown to repress transcription of the *cys *genes in that organism [1]. Finally, the co-regulation of *fpr2 *and the *cys *genes in *C. glutamicum *also provides circumstantial evidence that Fpr2 is involved in the reduction of sulphate.

The strong regulation of the three genes under study raised the question at which concentration it would occur. Therefore, the relative transcription levels in the presence of varying amounts of sulphate were measured. By this approach it could be demonstrated that already at a concentration of 100 *μ*M sulphate the genes are as strongly repressed as in the presence of 1 mM sulphate and more while at 50 *μ*M sulphate transcription levels are comparable to those observed in MMS without added sulphur (Fig. 5B).

### Determination of the transcription start sites in front of the *Corynebacterium glutamicum fpr2* and *cysI* genes

**Figure 6:**
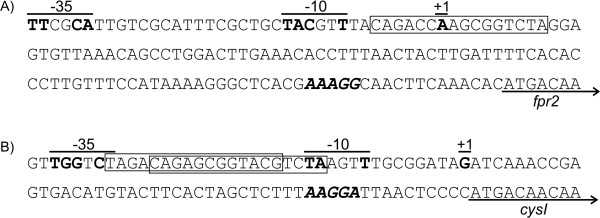
**The promoter/operator regions of the *C. glutamicum fpr2* and *cysI* genes**. The determined transcriptional start points of the two transcriptional units *fpr2 *(A) and *cysIXHDNYZ *(B) are marked as '+1'. Parts of the two potential promoters (-35, -10, +1) are overlined, bases in bold type in these regions indicate bases matching the *C. glutamicum *σ^70 ^consensus promoter [23]. DNA motifs matching the consensus sequence of the McbR binding-site are boxed. Bases in bold italics mark potential ribosome binding-sites, open underlining arrows indicate the annotated starts of genes.

As the real-time RT-PCR data indicated that all seven *cys *genes are described from one promoter, the RACE technique was applied to identify the promoter elements in the intergenic region between *fpr2 *and *cysI*. Thereby, one transcription start point was identified for each of the two transcription units (Fig. 6). The sequences upstream were compared to the the σ^70 ^consensus promoter [23] revealing two hexamers showing similarity to the -10 and -35 region including an optimal spacing of 17 bases, but no elements of an extended -10 region are detectable (Fig. 6). These hexamers are in 7 respectively 8 of 12 positions identical to the consensus sequence, which represents an average value for promoters in *C. glutamicum*, with the most conserved bases of the consensus promoter also present in the two promoters described here. An interesting finding was the observation that both promoters overlap with potential binding-sites of the transcriptional repressor McbR [15]. This strongly indicates that McbR is involved in the observed regulation.

## Discussion

In this report, we describe the identification and validation of a set of genes involved in the assimilatory reduction of sulphate in *Corynebacterium glutamicum*, including the up to now unknown genes *cysX, cysY, cysZ*, and *fpr2*.

**Figure 7:**
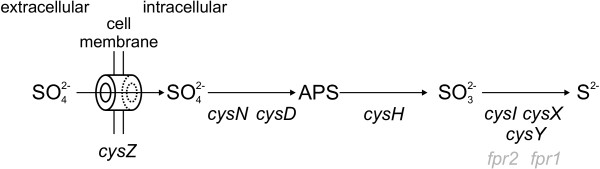
**Model of the pathway for assimilatory sulphur reduction in *C. glutamicum*.**. For proteins with gene names given in black, the involvement in the reduction of sulphate has been verified experimentally, for those in grey it has been inferred from circumstantial evidence.

Initial comparison of the genetic equipment for the assimilatory reduction of sulphate indicated that *C. glutamicum *possesses a set of genes similar to that known from *E. coli *[1], with exception of *cysC *(encoding APS kinase) and *cysJ *(encoding the flavoprotein subunit of sulphite reductase). On the other hand, genes typical for *B. subtilis *like *sat *[3], *ylnF *[7], and *cysP *[3] could not be found in *C. glutamicum*. Despite several similarities, the pathway present in *C. glutamicum *displays several features that distinguish it from that described for *E. coli*, based on the obtained data (Fig. 7):

Uptake of sulphate is most likely mediated by a novel type of permease, CysZ, instead of the ABC-type transporter Sbp CysPTWA known from *E. coli *[24,25]. Although not proven by biochemical data, the observed growth deficits of the Δ*cysZ *mutant strain together with the bioinformatic evidence that CysZ is located in the membrane strongly support this theory. Interestingly, the obtained data also indicates the existence of at least one low-affinity sulphate transporter and one or more transporter(s) for the uptake of the other inorganic sulphur compounds (like thiosulphate) which are not clustered with the other *cys *genes in *C. glutamicum*.

Strong evidence also exists that the activation of sulphate and the subsequent reduction to sulphite is performed in only two steps in *C. glutamicum*: Like *cysH *from *M. tuberculosis *[2], the heterologous expression of *cysH *from *C. glutamicum *can complement *E. coli cysH*^- ^as well as *cysC*^- ^mutants. This and the missing of a homologue of APS kinase clearly indicate that CysH acts as APS reductase in *C. glutamicum*, corroborating the prediction made by Lee [26].

The final step, the reduction of sulphite to sulphide seems to differ the most from the situation found in *E. coli *and *B. subtilis*, albeit this hypothesis is backed only by circumstantial evidence: CysI from *C. glutamicum *and the potential homologues in the other *Actinomycetales *share the highest degree of similarity to ferredoxin-dependent nitrite and sulphite reductases found in plants and cyanobacteria. Together with the presence of Fpr and CysX, this leads to the hypothesis that electrons are transferred from NADPH+H^+ ^via Fpr2 and CysX to CysI to reduce sulphite, similar to the pathway used in non-photosynthetic plant tissues [22]. Unfortunately, this hypothesis could not be proven conclusively with the used genetic methods alone. The loss of CysX clearly affected growth on all oxidised inorganic sulphur compounds, but the exact function cannot be inferred from this analysis. In case of Fpr2, the phenotypical evidence is even weaker as a mutant lacking Fpr2 is almost unaffected in the utilisation of sulphate and sulphite, except for a slightly increased lag phase. Still, the absence of a clear phenotype is easily explainable by the presence of a highly similar paralogous protein, Fpr1, that seems to be essential. The notion that Fpr2 is indeed involved in the pathway under study is also supported by the finding that it is co-regulated with the genes of the *cys *operon and is controlled by McbR, the global regulator of sulphur metabolism in *C. glutamicum *[15].

Based on the phenotype of the deletion mutant, CysY is also involved in the reduction of sulphite. The weak similarity of CysY to sirohydrochlorin ferrochelatase from *B. megaterium *(EC 4.99.1.4) [27] indicates an involvement in the biosynthesis of the siroheme cofactor of sulphite reductase, but again this assumption cannot be proven conclusively by genetic analysis alone.

Concerning the pathway as a whole, the data from the mutational analysis, i.e. the inability of *cysH, cysD*, and *cysN *mutants to utilise sulphite, and the failed heterologous complementation of *E. coli cys *mutants, suggest that the enzymes might form a multi-enzyme complex in *C. glutamicum*.

By analysing the mRNA levels of the eight clustered genes, it could be proven that *cysIXHDNYZ *constitute one single transcription unit. This unit seems to be co-regulated with *fpr2*, as expression of both was found to be strongly influenced to the same extent by the presence of most inorganic sulphur sources and L-cysteine.

The observed reduction of the mRNA levels of the eight genes under study in presence of various sulphur sources is reminiscent of the regulation described for *E. coli *[1] with exception for thiosulphate which also causes a strong anti-induction in *E. coli*. For *C. glutamicum*, it remains to be determined if the observed regulation is an anti-activation, as in *E. coli*, or a repression of a sulphur-starvation response. Based on the gathered data, the latter appears to be more likely, as *cysI *has been shown to be regulated by McbR, the global repressor of sulphur metabolism [28]. Mapping of the potential promoters of *cysI *and *fpr2 *revealed that both overlap with potential binding-sites of this master regulator [15], which corroborates the finding of Rey *et al*. that the transcription of the genes of the cluster is increased in a Δ*mcbR *mutant [15]. This strongly suggests that the observed regulation is at least in part due to the action of McbR. This hypothesis is further backed by the finding that McbR was found to be conserved in all completely sequenced *Corynebacteriaceae *[29] which is in accordance with the high degree of conservation of the cluster in *C. glutamicum, C. efficiens*, and *C. jeikeium*. Other candidates that might be involved in the regulation of the cluster are two genes under McbR-control, *cg0012 *and *cg0156*. The corresponding proteins both belong to the ROK/NagC-type family of regulators [29] and *cg0012 *has recently been proven to be involved in the metabolism of sulphur as it encodes the sulphate-inhibited activator of sulphonate utilisation, SsuR [30]. Therefore, *cg0156 *is a interesting candidate encoding a possible regulatory protein that should be characterised in a future study.

Although the regulatory protein(s) involved are not yet known, the regulatory network has been demonstrated to act extremely sensitive, as an external sulphate concentration of only 100 *μ*M already leads to a strong reduction of the mRNA levels of the *fpr2 *and *cys *genes by almost 1000-fold. This might indicate that the cell needs to strictly control the amounts of reduced sulphur, which is plausible as most of these compounds are toxic.

An interesting finding was the high degree of conservation among *cys *gene clusters in different members of the *Actinomycetales*. With exception of several pathogens and commensals. As the pathway described for *C. glutamicum *in this study seems to be present, with slight deviations, in at least 11 other species, it stands to argue that the findings for one member of this order can be easily transferred to other members. As *C. glutamicum *is one of the few non-pathogenic species of the *Actinomycetales*, and the genes, with exception of *fpr*, are present in only one copy, this bacterium is well suited to study this important pathway more closely, e.g. on the biochemical level.

## Conclusion

Based on sequence similarity, comparative genomics, and subsequent mutational analysis, we were able to identify a cluster of eight genes involved in assimilatory sulphate reduction. The obtained data supports a conclusive model for this pathway in *C. glutamicum *which differs considerably from those described for *E. coli *and *B. subtilis*, although further biochemical studies are necessary to prove the suggested functions. By using comparative genomics, we could gather strong evidence that the pathway described here might be present in at least 11 other members of the actinomycetes, thus distinguishing this group from the Gram-negative and low-GC Gram-positive bacteria in this metabolic function.

Furthermore, we could demonstrate that the eight genes under study are strongly regulated by the presence of either substrates and products of this pathway. Although reminiscent of the regulation described for *E. coli*, mapping of the transcription start points revealed that the *C. glutamicum *gene cluster might be controlled by repression of transcription rather than by anti-activation.

## Methods

### Bacterial strains, plasmids and culture media

The bacterial strains and plasmids used in this study are listed in Table 4. *E. coli *strains carrying plasmids were routinely grown on solid Antibiotic Medium No. 3 (PA) (Oxoid, Wesel, Germany) at 37°C. *C. glutamicum *strains were grown on solid brain-heart broth (BH) (VWR International, Darmstadt, Germany) at 30°C. Auxanography on solid medium was performed using a minimal medium (MMS), containing only trace amounts of inorganic sulphur. MMS is composed of 5% glucose, 150 mM NH_4_C1, 50 mM urea, 6 mM K_2_HP0_4_, 1.5 mM MgCl_2_, 70 *μ*M FeCl_2_, 50 *μ*M MnCl_2_, 70 *μ*M CaCl_2_, 7.5 *μ*M ZnCl_2_, 1 *μ*M CuCl_2_, 0.1 *μ*M, NiCl_2_, 500 *μ*g/l thiamine, and 50 *μ*g/l biotin, for the growth test different sulphur sources were added at 2 mM concentration (calculated for sulphur). For solidification, 16 g agarose were added per litre MMS.

For growth tests in liquid medium, a modified version of MME [31], called MMES, was used, consisting of 2.5% glucose, 17 mM NaNH_4_HP0_4_, 1 mM MgCl_2_, 60 mM K_2_HP0_4_, 10 mM citric acid, 37.5 *μ*M FeCl_2_, 50 *μ*M MnCl_2_, 67.5 *μ*M CaCl_2_, 7.5 *μ*M ZnCl_2_, 1 *μ*M CuCl_2_, 0.1 *μ*M, NiC1_2_, 500 *μ*g/l thiamine, and 50 *μ*g/l biotin, with addition of different sulphur sources at varying concentrations.

Sulphur sources used were sodium sulphate (Na_2_S0_4_), sodium sulphite (Na_2_SO_3_), sodium thiosulphate (Na_2_S_2_O_3_), sodium sulphide (Na_2_S), and L-cysteine.

Antibiotics used for selection of plasmids and strains were nalidixic acid (50 *μ*g/ml for corynebacteria) and kanamycin (50 *μ*g/ml for *E. coli*, 25 *μ*g/ml for corynebacteria).

### DNA isolation, transfer and manipulation

Standard procedures were employed for molecular cloning and transformation of *E. coli *DH5α, as well as for electrophoresis [32]. Transformation of *C. glutamicum *was performed by electroporation using the methods of Tauch *et al*. [33].

### Construction of plasmids

Plasmids pCR016d to pCR025d were constructed using the gene splicing by overlap extension (gene-SOEing) method described by Horton *et al*. [34], the PCR primers used are listed in the supplementary Table S1 [see Additional file 1]. The primary products were amplified using *Pwo *DNA polymerase (Roche, Mannheim, Germany). The resulting products were purified using the PCR purification kit (QIAGEN, Hilden, Germany) and used as templates for the second round of PCR. The final products were digested with restriction enzymes corresponding to the cleavage sites introduced via PCR and ligated into appropriately digested pK18*mobsacB*. The ligation mixture was used to transform *E. coli *DH5αMCR, the transformants were selected on PA plates containing 50 *μ*g/ml kanamycin and 40 mg/l X-Gal (5-bromo-4-chloro-3-indolyl-β-D-galactopyranoside).

Plasmids for complementation experiments were created by amplifying the gene of interest via PCR. The resulting constructs were cleaved using restriction sites added by the PCR primers and then ligated into plasmid pZ8-l, cut with the corresponding enzymes. The ligation mixture was used for transformation of *E. coli *DH5αMCR, the transformants were selected on PA plates containing 50 *μ*g/ml kanamycin.

### Site-specific gene deletion

Site-specific gene deletion was performed using the non-replicable integration vector pK18*mobsacB *which allows for marker-free deletion of the target gene [35]. The plasmids pCR018d to pCR025d were transferred into the *C. glutamicum *wild-type strain (ATCC 13032) by electroporation [33]. Tests for first and second cross-over were performed as described previously [8].

### Identification of a cysI transposon mutant

Using a cloned copy of the IS*6100 *mobile element [36], a transposon mutant library was constructed. The mutants were gained with a modified approach that uses electrotransfer of the IS*6100*-based transposon vector pAT6100 into the *C. glutamicum *host cells. Subsequent phenotypic screening of the transposon library for cystein-auxotrophic mutants was applied, and the transposon integration loci of these mutants were determined by a plasmid rescue technique and subsequent sequence similarity analysis [37].

### Relative quantification of mRNA levels using real-time RT-PCR

Bacterial cell cultures were grown to the early logarithmic phase (o.D._600 _between 8 to 12) in MMS with 250 *μ*M L-cysteine as sulphur source. Incubation took place in an Innova 4430 orbital shaker (New Brunswick, N J) at 300 rpm, using 25 ml medium in a 250 ml Erlenmeyer flask.

For each experiment, 5*10^9 ^cells were pelletised by centrifugation, washed twice with MMS without added sulphur source (preheated to 30°C) and resuspended in 5 ml MMS. To this culture the sulphur source to be analysed was added at varying concentrations and incubated for an additional 30 min.

For RNA isolation, about 10^9 ^cells per culture were harvested by 15 sec centrifugation at 16,000 g, followed by immediate removal of the supernatant and freezing of the pellet in liquid nitrogen. Preparation of total RNA from *C. glutamicum *cells was performed as described by Hüser *et al*. [38].

Primers for real-time RT-PCR were constructed to amplify intergenic regions of about 150 bp length of the genes to be analysed, designated as *_lc1/2 [see Additional file 1]. The primers were designed using the Primer Designer 4.2 software (Sci Ed Software, Durham, NC) and were purchased from SIGMA-ARK (Darmstadt, Germany).

All real-time RT-PCR experiments were performed using a LightCycler (Roche, Mannheim, Germany) with the QuantiTect SYBR Green RT-PCR Kit (QIAGEN, Hilden, Germany). PCR mixes were set up and PCR was performed as described by Koch *et al*. [39]. All measurements were performed for two biological replicates per condition tested and with two technical replicates per biological replicate. The amounts of the mRNAs of the genes of the cluster were normalised on total RNA and the relative change in transcription rate was determined as 2^-Δ*CP *^with ΔCP equal to the difference of the measured crossing points for the test and the control condition.

### Determination of transcriptional starts with the RACE method

Total RNA was isolated from a *C. glutamicum *wild-type culture grown in MMS medium and subjected to sulphur starvation as described above. Primers binding downstream of the annotated translational starts of the *cysI *and *fpr2 *genes (*SP1-3 in Table 5) along with 1.5 *μ*g of total RNA were used for cDNA synthesis. The cDNA was then modified and amplified using the 5'/3' RACE kit (Roche Diagnostics) according to the supplier's protocol. The obtained PCR products were cloned into the pCR-Blunt II-TOPO vector (Invitrogen, Karlsruhe, Germany) and transferred into *E. coli *DH10B cells [40]. At least four different clones per gene were selected for plasmid preparation and DNA sequencing (IIT Biotech, Bielefeld, Germany).

### Real-time monitoring of cell growth using nephelometry

All strains to be tested (the wild-type strain and different mutants) were grown overnight in liquid BH medium in an Innova 4430 orbital shaker (New Brunswick, NJ) at 300 rpm. One ml of an o/n culture was pelletised, washed once with liquid MMES and transferred into a 100 ml Erlenmeyer flask containing 10 ml fresh, preheated MMES with addition of 500 *μ*M L-cysteine as sulphur source. After o/n incubation in an orbital shaker at 300 rpm, one ml of the culture was again washed and transferred into 10 ml MMES as described above. These cultures were incubated for 6 hours in an orbital shaker at 300 rpm.

For real-time growth monitoring, 1 ml of each culture was pelletised and washed twice with MMES. For each test condition, 1 ml MMES with addition of a sulphur source at varying concentrations was inoculated with washed cells of the strain to be tested for a final o.D._600 _of 0.01. Per test condition, 6 wells of a 96-well Cellstar suspension culture plate (Greiner Bio-One, Essen, Germany) were filled with 100 *μ*l inoculated MMES per well. The plates were sealed with Breathe-Easy membrane (Diversified Biotech, Boston, MA) and growth was measured in a microplate nephelometer (BMG Lab Technologies, Offenburg, Germany). For these measurements, the parameters selected were: Incubation at 30°C, 50% laser intensity, gain of 86, 2.5 mm laser focus point, 900 sec orbital shaking with 3 mm shaking width, and measurement after each cycle with 0.2 sec measurement time per well, 0.0 sec positioning delay.

### GenBank/TrEMBL accession numbers

All protein sequences from other organisms were obtained from the UniProt database [41]. The nucleotide sequences of all genes from *C. glutamicum *mentioned can be found via the genome entry (accession number [EMBL:BX927147]) with the ORF name as locus-tag. The amino acid sequences of the corresponding proteins are available under the following accession numbers: Cg3049 [EMBL:CAF20776], Cg3112 [EMBL:CAF20834], Cg3113 [EMBL:CAF20835], Cg3114 [EMBL:CAF20836], Cg3115 [EMBL:CAF20837], Cg3116 [EMBL:CAF20838], Cg3117 [EMBL:CAF20839], Cg3118 [EMBL:CAF20840], and Cg3119 [EMBL:CAF20841]. The accession numbers of the genome sequences used for comparative genomics are [EMBL:AE014295] (*Bifidobacterium longum*), [EMBL:BX248353] (*C. diphtheriae*), [EMBL:BA000035] (*C. efficiens*), [EMBL: AE016822] (*Leifsonia xyli*), [EMBL:AE016958] (*Mycobacterium avium*), [EMBL:AL450380] (M. *leprae*), [EMBL:AL123456] (M. *tuberculosis*), [EMBL:AP006618] (*Nocardia farcinica*), [EMBL:AE017283] (*Propionibacterium acnes*), [EMBL:BA000030] (*Streptomyces avermitilis*), [EMBL:AL645882] (*S. coelicolor*), and [EMBL:BX072543] (*Tropheryma whipplei*).

### Bioinformatic analysis

Sequence similarity-based searches with nucleotide and protein sequences were performed using BLAST, the Basic Local Alignment Search Tool [42] against the UniProt database [41]. Searches using profile Hidden Markov Models (HMMs) from the PFAM database [43] were done using the HMMer software package. Transmembrane domains were searched for using the TMHMM software [44] while for the detection of signal peptides SignalP [45] was used. Multiple alignments were done using DIALIGN 2 [46]. Search for conserved gene clusters was performed using the GECKO software [19] and TheSEED [20].

## Authors' contributions

CR performed the bioinformatic analyses and carried out the mutational and transcriptional studies. DJK aided the mutational analysis and performed additional growth tests. DAR carried out transcriptional studies. AA performed the mapping of transcriptional start points. SM constructed the transposon library and identified a suitable mutant. AP aided in coordination and conceived of the design of tables and figures. JK conceived and coordinated this study. All authors read and approved of the final manuscript.

## Supplementary Material

Additional File 1 **Supplementary Table S1.eps **Supplementary Table S1 Oligonucleotides used in this study. Click here for file
